# Supplementary material for: Design, Synthesis and Mechanistic Studies of Novel Isatin-Pyrazole Hydrazone Conjugates as Selective and Potent Bacterial MetAP Inhibitors
Source: Antibiotics (Basel). 2022 Aug 19;11(8):1126. doi: 10.3390/antibiotics11081126 (PMC9405123; doi:10.3390/antibiotics11081126)
Supplement: Supplementary file 1 [file antibiotics-11-01126-s001.zip › antibiotics-1874232-supplementary.pdf]

Design, synthesis and mechanistic studies of novel Isatin-pyrazole hydrazone conjugates as selective and potent bacterial MetAP Inhibitors

Table of contents

	Contents	Page No.
1.	Spectral data of all compounds (PS1-14)	S3-29
2.	Table S1: Detail of environmental bacterial isolates	S30-30
3.	Table S2: Zone of inhibition (mm) measured around the disk of various concentration of the compounds	S31-31
4.	Physiochemical properties of the compounds	S32-32
5.	Morrison Ki plots for MetAP against Isatin-pyrazole hydrazone conjugates	S33-35

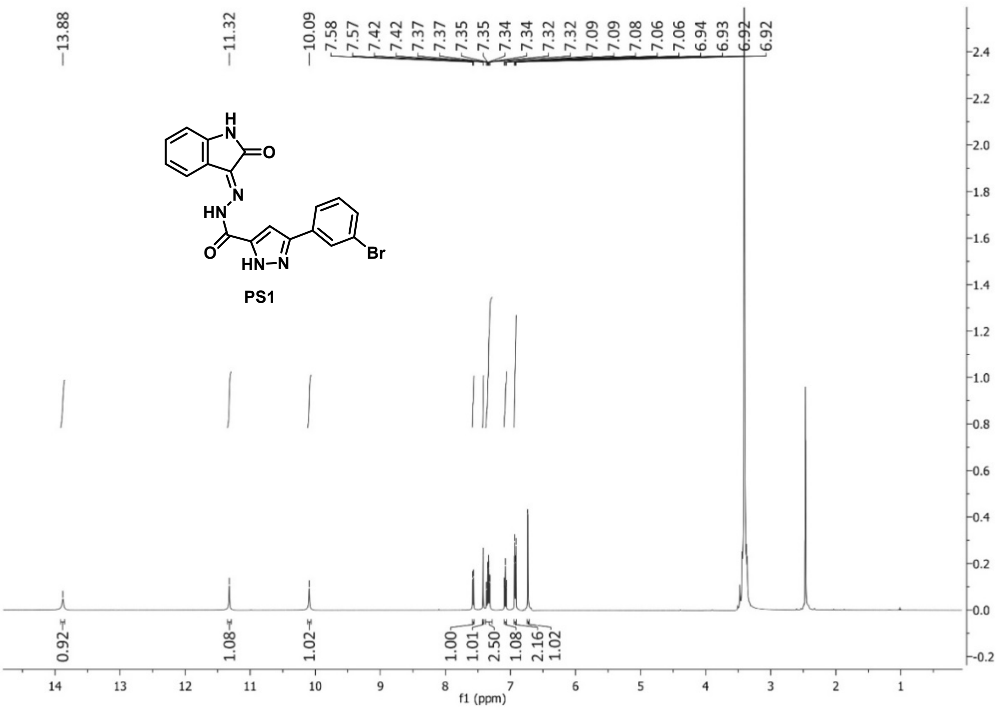

[1]H of compound PS1

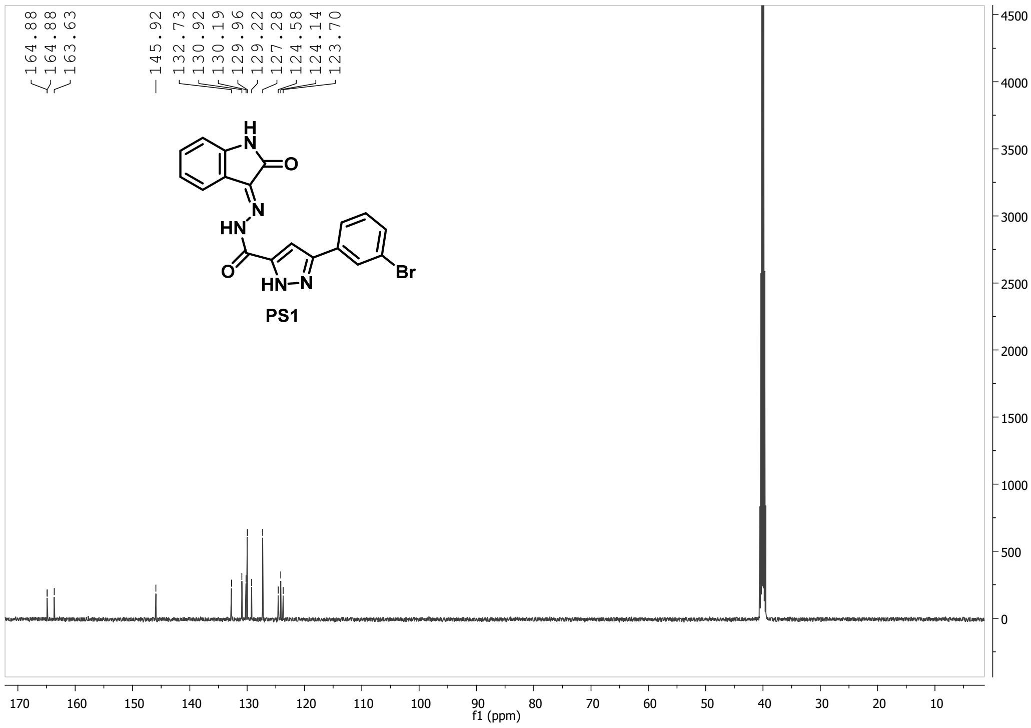

[13]C of compound PS1

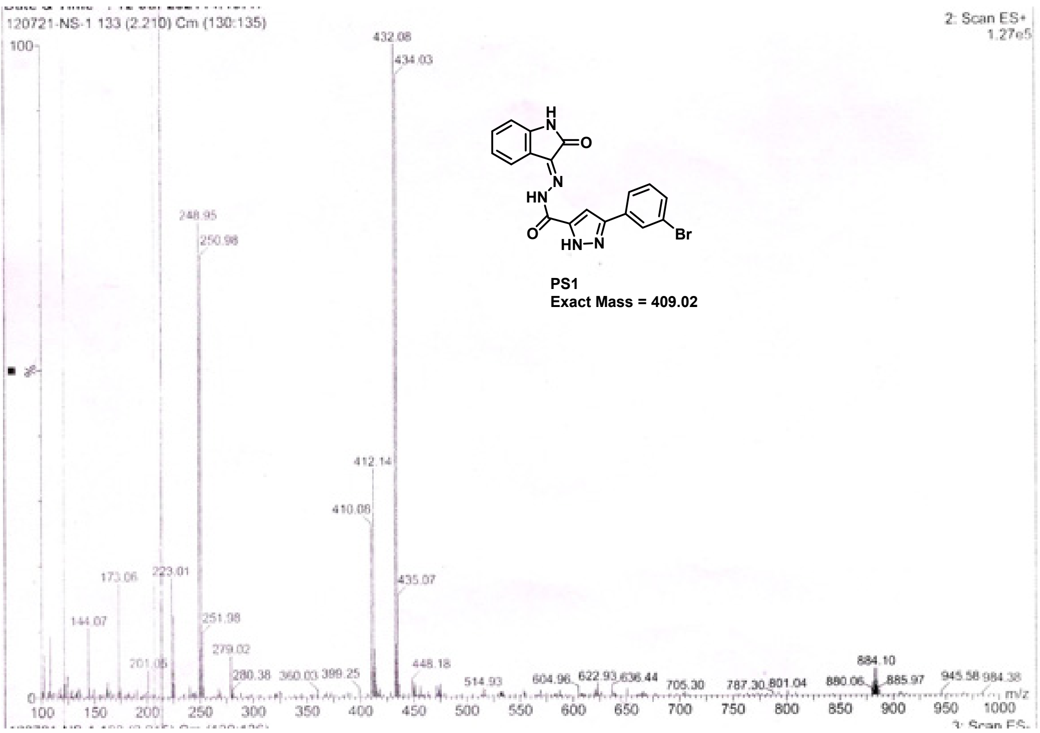

Mass spectra of compound PS1

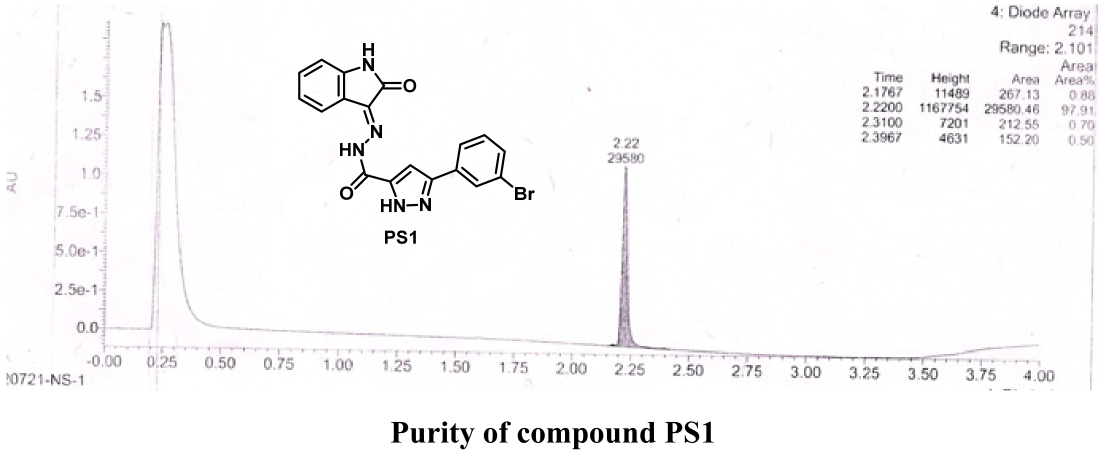

Purity of compound PS1

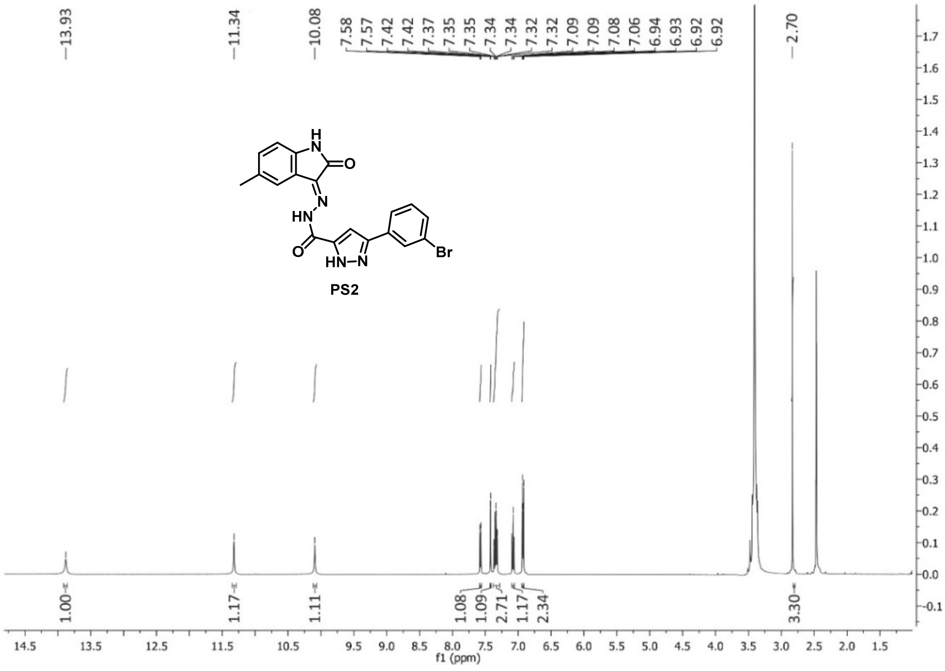

^{1}H of compound PS2

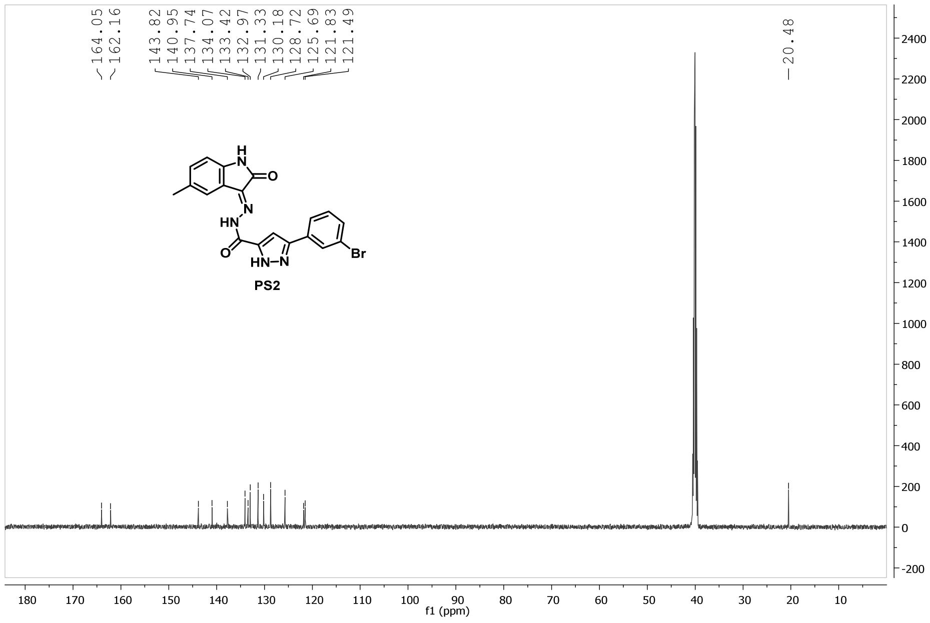

^{13}C of compound PS2

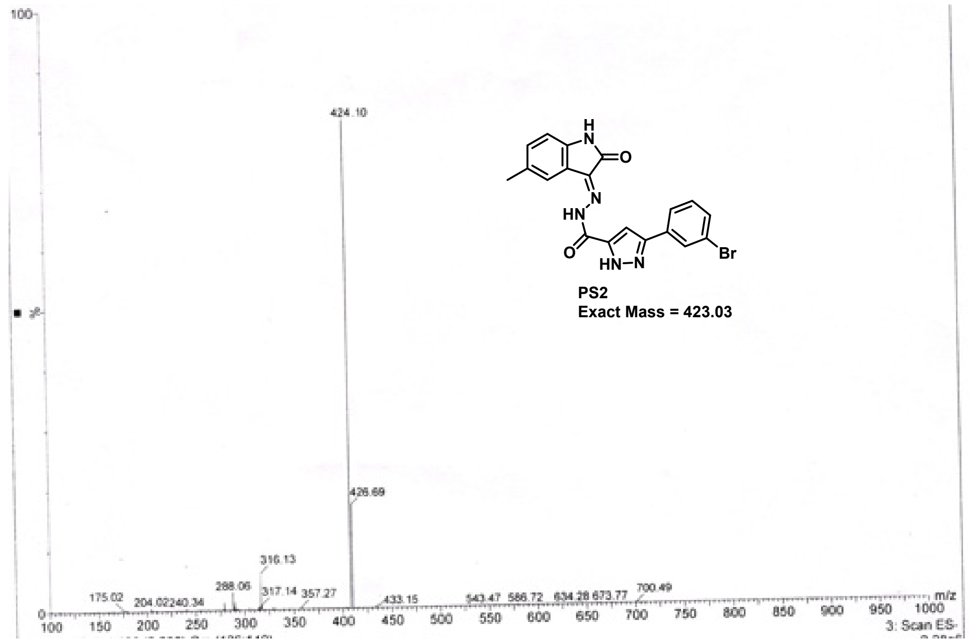

Mass spectra of compound PS2

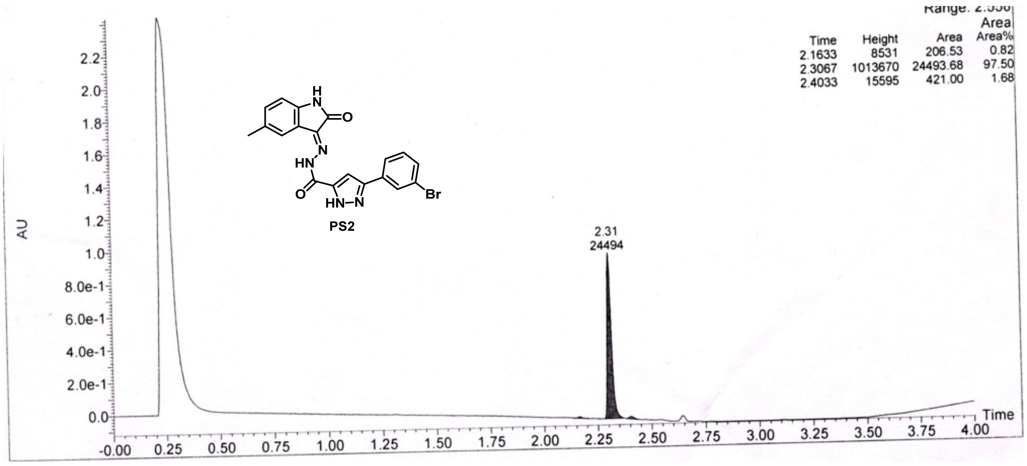

Purity of compound PS2

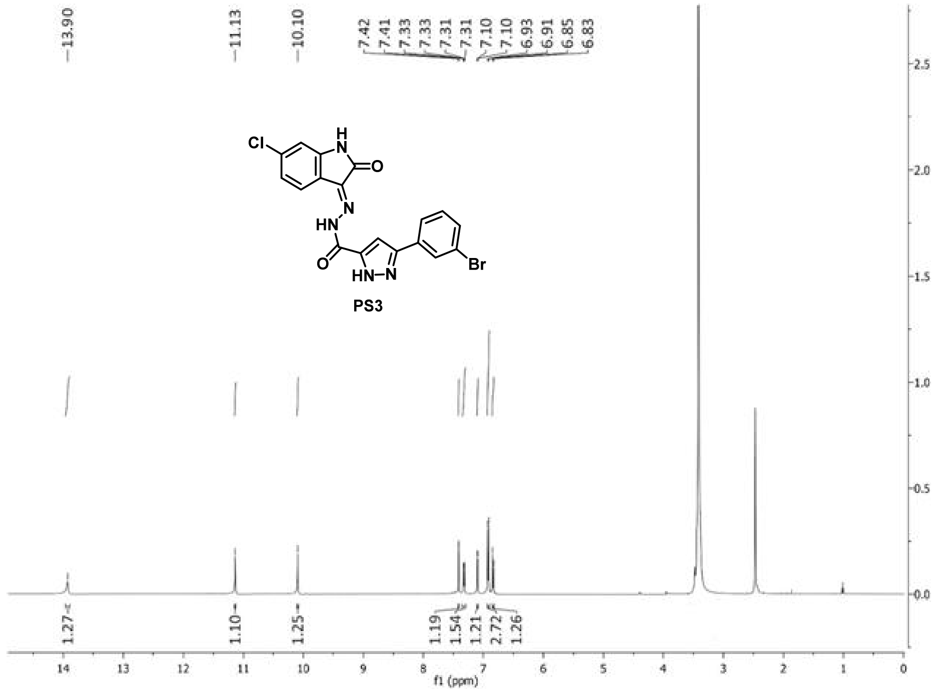

[1]H of compound PS3

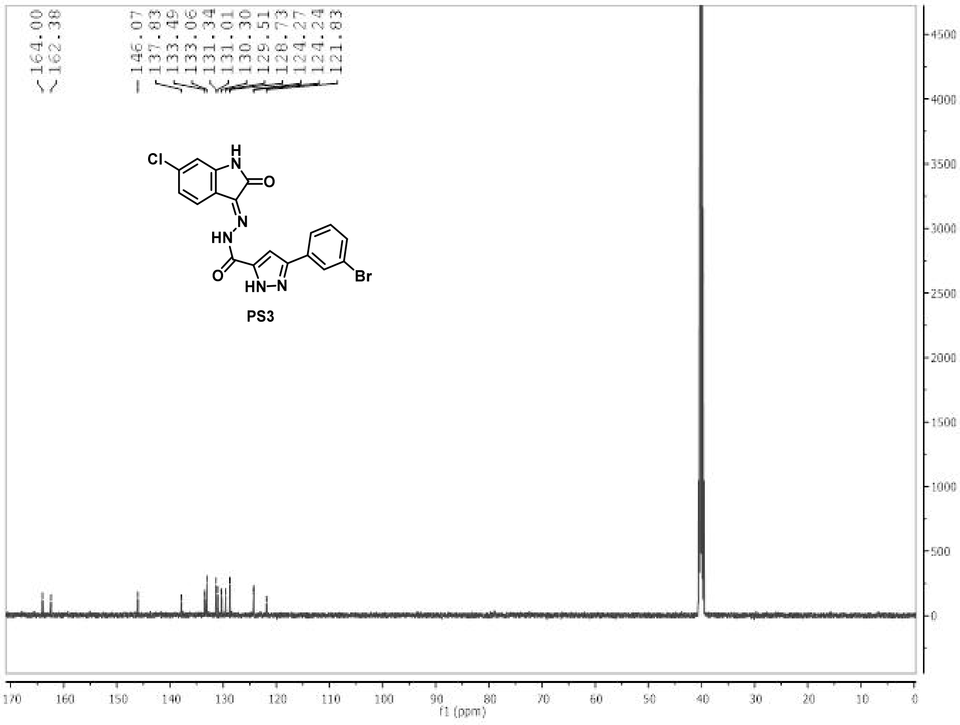

[13]C of compound PS3

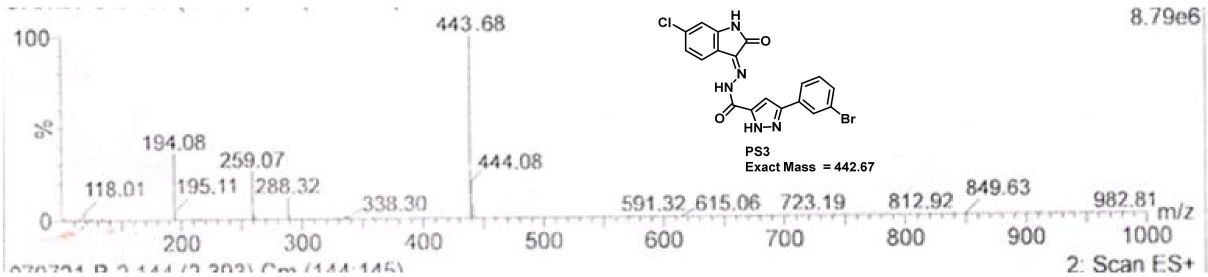

Mass spectra of compound PS3

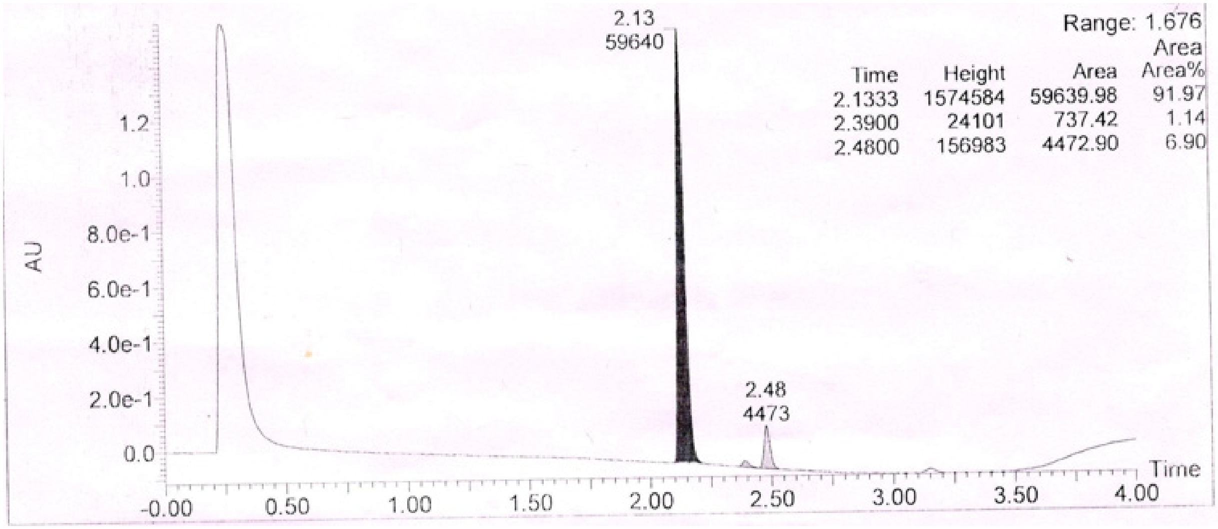

Purity of compound PS3

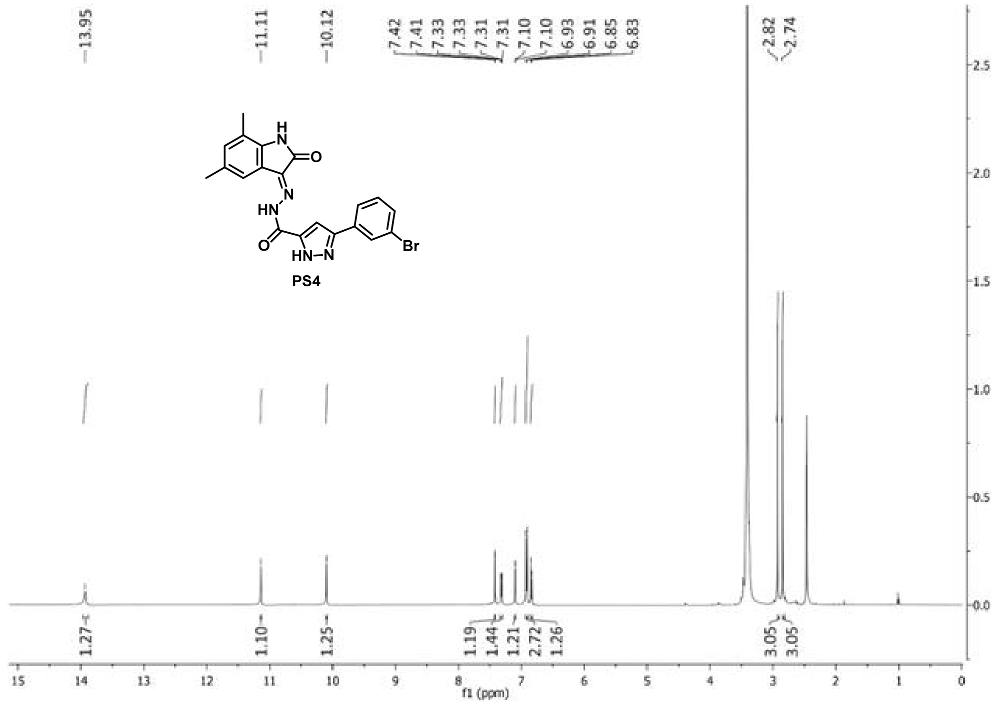

^{1}H of compound PS4

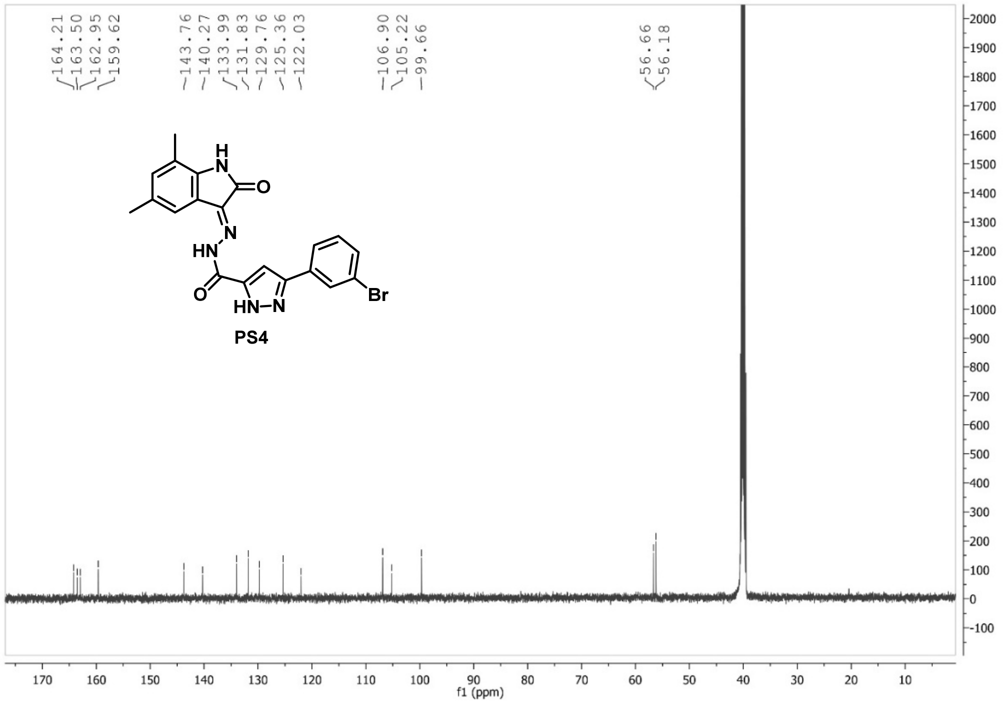

^{13}C of compound PS4
